# Short‐term disuse does not affect postabsorptive or postprandial muscle protein fractional breakdown rates

**DOI:** 10.1002/jcsm.13284

**Published:** 2023-07-11

**Authors:** George F. Pavis, Doaa R. Abdelrahman, Andrew J. Murton, Benjamin T. Wall, Francis B. Stephens, Marlou L. Dirks

**Affiliations:** ^1^ Nutritional Physiology Research Group, Public Health & Sport Sciences, Faculty of Health and Life Sciences University of Exeter Exeter UK; ^2^ Department of Surgery University of Texas Medical Branch Galveston TX USA; ^3^ Sealy Center of Aging University of Texas Medical Branch Galveston TX USA; ^4^ Human and Animal Physiology Wageningen University Wageningen The Netherlands

**Keywords:** Amino acids, Immobilization, Muscle disuse atrophy, Muscle protein breakdown, Protein synthesis

## Abstract

**Background:**

The decline in postabsorptive and postprandial muscle protein fractional synthesis rates (FSR) does not quantitatively account for muscle atrophy during uncomplicated, short‐term disuse, when atrophy rates are the highest. We sought to determine whether 2 days of unilateral knee immobilization affects mixed muscle protein fractional breakdown rates (FBR) during postabsorptive and simulated postprandial conditions.

**Methods:**

Twenty‐three healthy, male participants (age: 22 ± 1 year; height: 179 ± 1 cm; body mass: 73.4 ± 1.5 kg; body mass index 22.8 ± 0.5 kg·m^−2^) took part in this randomized, controlled study. After 48 h of unilateral knee immobilization, primed continuous intravenous l‐[^15^N]‐phenylalanine and l‐[*ring*‐^2^H_5_]‐phenylalanine infusions were used for parallel determinations of FBR and FSR, respectively, in a postabsorptive (saline infusion; FAST) or simulated postprandial state (67.5 mg·kg body mass^−1^·h^−1^ amino acid infusion; FED). Bilateral *m. vastus lateralis* biopsies from the control (CON) and immobilized (IMM) legs, and arterialized‐venous blood samples, were collected throughout.

**Results:**

Amino acid infusion rapidly increased plasma phenylalanine (59 ± 9%), leucine (76 ± 5%), isoleucine (109 ± 7%) and valine (42 ± 4%) concentrations in FED only (all *P* < 0.001), which was sustained for the remainder of infusion. Serum insulin concentrations peaked at 21.8 ± 2.2 mU·L^−1^ at 15 min in FED only (*P* < 0.001) and were 60% greater in FED than FAST (*P* < 0.01). Immobilization did not influence FBR in either FAST (CON: 0.150 ± 0.018; IMM: 0.143 ± 0.017%·h^−1^) or FED (CON: 0.134 ± 0.012; IMM: 0.160 ± 0.018%·h^−1^; all effects *P* > 0.05). However, immobilization decreased FSR (*P* < 0.05) in both FAST (0.071 ± 0.004 vs. 0.086 ± 0.007%·h^−1^; IMM vs CON, respectively) and FED (0.066 ± 0.016 vs. 0.119 ± 0.016%·h^−1^; IMM vs CON, respectively). Consequently, immobilization decreased net muscle protein balance (*P* < 0.05) and to a greater extent in FED (CON: −0.012 ± 0.025; IMM: −0.095 ± 0.023%·h^−1^; *P* < 0.05) than FAST (CON: −0.064 ± 0.020; IMM: −0.072 ± 0.017%·h^−1^).

**Conclusions:**

We conclude that merely 2 days of leg immobilization does not modulate postabsorptive and simulated postprandial muscle protein breakdown rates. Instead, under these conditions the muscle negative muscle protein balance associated with brief periods of experimental disuse is driven near exclusively by reduced basal muscle protein synthesis rates and anabolic resistance to amino acid administration.

## Introduction

Skeletal muscle disuse, which is often experienced during illness or injury, leads to rapid and substantial muscle atrophy of approximately 0.5–0.8% per day.^1^, ^2^ This muscle disuse atrophy forms a significant health concern, with both bed rest^3^, ^4^ and limb immobilization^5^, ^6^, ^7^ models of disuse revealing an associated decline in muscle strength and insulin sensitivity. However, the underlying mechanisms remain to be fully understood.

Muscle disuse atrophy is underpinned by sustained negative muscle protein balance, which must arise through reduced rates of muscle protein synthesis (MPS), increased rates of muscle protein breakdown (MPB), or a combination of both. Longer term (>14 days) periods of disuse result in 10–50% reductions in MPS, without concurrent changes in MPB.^8^, ^9^, ^10^, ^11^ The decline in postabsorptive MPS and blunting of MPS in the postprandial state, in a phenomenon termed ‘anabolic resistance’, are widely considered to be the primary causative mechanisms of disuse atrophy.^5^, ^10^, ^12^ In support, our recent work demonstrated that the decrease in daily MPS rates, which capture multiple postabsorptive and postprandial periods, positively correlate with reductions in muscle volume after 7 days of unilateral leg immobilization (*r* ^2^ = 0.417, *P* < 0.05).^13^ It is therefore suggested that this decline in MPS quantitatively accounts for only ~25–50% of the measured muscle loss with short‐term (<14 days) disuse^13^, ^14^ when the rate of atrophy is greatest,^15^, ^16^ although some work has argued MPS can potentially explain an even greater proportion.^12^, ^17^ Moreover, marked reductions in muscle volume are evident after only 2 days of immobilization (i.e., ~0.85% per day^18^) despite less a pronounced decline in daily MPS^13^ and can occur in the absence of any changes in MPS if high‐volume and high‐intensity eccentric contractions are performed before immobilization.^19^ As such, a significant portion of early disuse atrophy remains mechanistically unexplained.

The fractional synthetic rate (FSR) of muscle protein is widely used to quantify MPS directly from the incorporation of an isotopically‐labelled amino acid tracer into muscle protein. Direct and dynamic measurements of MPB (i.e., fractional breakdown rate, FBR) have been performed in humans following resistance exercise^20^, ^21^ and burn injury,^22^ and validated against arterial–venous balance models,^23^ but despite the comprehensive nature of the measurement and insight it provides with regard to muscle protein turnover, it has not been conducted following short‐term disuse. Nonetheless, static molecular markers (i.e., gene expression of ubiquitin‐proteasome components, MuRF1 and MAFBx) are consistent with a rise of MPB after 2^7^, ^24^ and 5 days,^25^ but not 14 days of immobilization.^7^, ^25^ Recently, more dynamic measurements have been made through characterizing the dilution rate of an amino acid tracer in the muscle intracellular pool.^17^ The dilution rate, of which MPB is one of several contributing factors, was comparable between a control and immobilized leg. However, without determining FBR, it remains unknown how disuse affects MPB.^17^ Moreover, dilutions were only measured in the postabsorptive state, and MPB is generally thought to be suppressed by protein‐rich food intake,^1^ thereby contributing to the overall anabolic response. Given the reduced sensitivity of MPS to feeding following immobilization, it is plausible that MPB may be similarly less responsive and thereby further contribute to early muscle disuse atrophy. However, no work to date has addressed how MPB changes under postprandial conditions with immobilization.

In the present study, we used tracer dilution and incorporation principles^23^ to directly determine the effect of 2 days of one‐legged knee immobilization on MPB and MPS rates, measured simultaneously, for the first time. This was performed in healthy young males in the postabsorptive state and separately during intravenous amino acid infusions to simulate a postprandial condition. We hypothesized that short‐term immobilization would increase postabsorptive and simulated postprandial rates of MPB, contributing to a negative net muscle protein balance in both states.

## Methods

### Participants

Twenty‐three healthy males (age: 22 ± 1 year; height: 179.4 ± 1.3 cm; body mass: 73.4 ± 1.5 kg; body mass index 22.8 ± 0.5 kg·m^−2^) volunteered to take part in this study. Prior to inclusion, participants attended the Nutritional Physiology Research Unit at the University of Exeter for routine medical screening to ensure they did not contravene the following exclusion criteria: diagnosed metabolic impairment (e.g., type 1 or type 2 diabetes); cardiovascular disease; hypertensive (≥140/90 mmHg); chronic use of prescribed or over‐the‐counter pharmaceuticals; personal or family history of epilepsy, schizophrenia, or seizures; presence of an ulcer in the stomach or gut; pre‐existing liver or kidney condition; musculoskeletal injury within 6 months prior to enrolment; having previously received a stable isotope tracer containing ^15^N; regular use of nutritional supplements; and/or an allergy or intolerance to lidocaine or amino acids. Written consent was obtained from all participants following full explanation of the experimental procedures, which were approved by the University of Exeter's Sport and Health Sciences Ethics Committee (180509/B/01). This study was registered at ClinicalTrials.gov as NCT03934632.

### Experimental protocol

**Figure 1:**
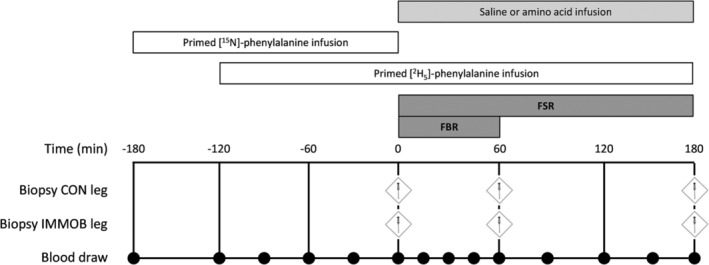
Graphical representation of the experimental test visit performed at 48 h of single leg immobilization. Fractional breakdown rate (FBR) measured between 0–60 min; fractional synthesis rate (FSR) measured between 0, 60, and 180 min; of intravenous saline (FAST; *n* = 11) and amino acid (FED; *n* = 12) infusion.

Following inclusion, participants were randomly assigned to either a postabsorptive (FAST) or simulated postprandial (FED) group in a single‐blind, parallel group manner. Exactly 48 h prior to the start of a single experimental test day, participants visited the Nutritional Physiology Research Unit at 8:00 am for unilateral knee immobilization. The leg randomized to undergo immobilization (IMM) was counterbalanced within each group for leg dominance, while the contralateral leg served as a free‐living, within‐subject control (CON). It is well established that muscle volume and/or cross‐sectional area of the non‐immobilized control leg remains unchanged during one‐legged knee immobilization lasting 2–14 days,^12^, ^18^, ^26^, ^27^, ^28^ suggesting that muscle protein net balance is unaltered, making this an appropriate internal control. With the IMM knee immobilized at 70° flexion (full knee extension considered as 0°; see supporting information), participants were instructed how to use crutches and asked to refrain from bearing any weight on the immobilized leg, as well as to refrain from alcohol and strenuous exercise during immobilization. After consuming a standardized meal by 10:00 pm the prior evening (see supporting information), participants returned by taxi to the Nutritional Physiology Research Unit at 8:00 am on the morning of the test day (Figure 1). A venous blood sample was obtained to measure background isotope enrichments (*t* = −180 min), after which the plasma phenylalanine pool was primed with 0.5 mg·kg^−1^ body mass^−1^ l‐[^15^N]‐phenylalanine. Continuous venous infusion of l‐[^15^N]‐phenylalanine began thereafter at 0.5 mg·kg^−1^·h^−1^. Repeated arterialized venous blood samples were obtained from a heated, dorsal hand vein (55°C, and kept patent with a continuous 0.9% saline infusion), which provides an accurate reflection of arterial metabolite concentrations.^29^

Sixty min into the l‐[^15^N]‐phenylalanine infusion (*t* = −120 min) a primed‐continuous infusion of l‐[*ring*‐^2^H_5_]‐phenylalanine (priming dose 0.5 mg·kg^−1^; infusion rate 0.5 mg·kg^−1^·h^−1^) began and continued for the remainder of the experimental trial. At *t* = 0, bilateral biopsies of the *m. vastus lateralis* were obtained under local anaesthesia (2% lidocaine). The l‐[^15^N]‐phenylalanine infusion was then immediately stopped. For participants in FED, a primed infusion of amino acids (Primene 10%, Baxter Healthcare Ltd, Norfolk, UK) enriched to 7% with l‐[*ring*‐^2^H_5_]‐phenylalanine was simultaneously started (priming dose 23.0 mg·kg^−1^; infusion rate 67.5 mg·kg^−1^·h^−1^) and continued until the end of the testing protocol. Participants in FAST received a volume‐matched, primed‐continuous infusion of saline. Additional bilateral biopsies were obtained at *t* = 60 and 180 min from *m. vastus lateralis* ~2.5 cm proximal to the previous incision on each occasion. Biopsy samples were rapidly blotted to remove blood, dissected of visible fat and connective tissue, snap‐frozen in liquid nitrogen and stored at −80°C for further analysis.

### Plasma, intracellular, and mixed muscle amino acid analysis

Norleucine internal standard was added to plasma defrosted at room temperature, before being deproteinized with an equal volume of 15% sulphosalicylic acid (weight/vol %). Following centrifugation at 4000× *g* for 10 min at 4°C, the supernatant was passed over cation exchange resin columns (100–200 mesh; H^+^ form; AmberChrom 50WX8; Sigma‐Aldrich Company Ltd, Gillingham, UK). Columns were washed with 0.5 M acetic acid and ddH_2_O and eluted with 6 M ammonium hydroxide, before being dried under vacuum and stored at −20°C for later derivatization.

Amino acids from intracellular and mixed muscle protein‐bound pools were extracted from ~30 mg wet weight tissue. Samples were freeze dried for 50 h and powdered by hand. Muscle powder was freed from visible blood and connective tissue before being mixed with 500 μL 1 M ice‐cold perchloric acid and norleucine internal standard. Samples were kept on ice for 10 min and regularly vortexed, prior to centrifugation at 10 000 × *g* for 10 min at 4°C. Supernatants containing the intracellular free amino acid pool were aliquoted and neutralized with 1 M NaOH. The remaining protein pellet was washed once with 1 mL 70% ethanol and hydrolysed in 2 mL 6 M HCl with norleucine internal standard at 110°C for 24 h. To account for any lost protein, aliquots of the ethanol wash and neutralized IC pool were obtained and analysed for protein content by colorimetric assay (DC protein assay, Bio‐Rad Laboratories Ltd., Watford, UK). The hydrolysed pellets were subsequently dried under a vacuum and reconstituted in 3 mL acetic acid and passed over cation exchange resin columns. The neutralized supernatant aliquots containing intracellular free amino acids were also passed over cation exchange resin columns and washed with 3 mL acetic acid. All columns were rinsed with ddH_2_O and eluted and dried as described above.

All amino acid extracts were derivatized to their TBDMS‐esters and analysed by GC–MS (7890 GC coupled with a 5975 MSD; Agilent Technologies, Santa Clara, CA, USA) in duplicate using electron impact ionization and selected ion monitoring for measurement of isotope ratios. Mass ratios of m/z 336, 337, and 341 were selected to detect intracellular and plasma free phenylalanine isotopes. For the protein‐bound portion, the fragments m/z 237 and 239 were monitored and corrected against a standard curve. Plasma leucine, isoleucine, valine, phenylalanine, and tyrosine concentrations were calculated from the mass charge corresponding to the unlabelled ion relative to that of the internal standard. For all targets, a series of known standards was applied to assess linearity of the mass spectrometer.

### Calculations

We applied tracer‐dilution principles with a modified precursor‐product equation to calculate the fractional breakdown rate (FBR) of mixed muscle protein as follows:

(1) $$\mathrm{FBR}\;\left(\%\cdot h^{-1}\right)=\frac{E_{\mathrm{IC}2}-E_{\mathrm{IC}1}}{p\;\int_{t_{1}}^{t_{2}}E_{A\left(t\right)}\mathrm{dt}-\left(1+p\right)\int_{t_{1}}^{t_{2}}E_{\mathrm{IC}\left(t\right)}\mathrm{dt}}\times \frac{Q_{M}}{T}\times 100\%$$

where *E*_*A*_ and *E*_*IC*_ are the arterialized plasma and intracellular free enrichments, respectively, at time (*t*) of biopsy 1 (0 min) and biopsy 2 (60 min), *p = E*_*IC*_ */ (E*_*A*_ *– E*_*IC*_ *)* at isotopic steady state, and *Q*_*M*_ */T* is the ratio of IC to protein‐bound phenylalanine concentrations in muscle. The decay of arterialized plasma phenylalanine enrichment from *t*_*1*_ to *t*_*2*_ was fitted to a two‐phase exponential curve using MATLAB (version R2019a; MathWorks, Natick, MA, USA) and Ezyfit Toolbox (FAST, University Paris‐Sud, France). This permits FBR to be calculated using a single biopsy during isotopic decay (i.e., *t* = 60), as for a given value of IC phenylalanine enrichment during physiologic steady state there is only one FBR value.^23^

The fractional synthesis rate (FSR) of mixed muscle proteins was calculated from the incorporation of l‐[*ring*‐^2^H_5_]‐phenylalanine from either the arterialized or IC pools (i.e., precursor) into mixed muscle proteins (i.e., product) using the following equation:

(2) $$\mathrm{FSR}\;\left(\%\cdot h^{-1}\right)=\frac{E_{M2}-E_{M1}}{\int_{t_{1}}^{t_{2}}E_{P\left(t\right)}\mathrm{dt}}\times 100\%$$

where *E*_*M2*_ – *E*_*M1*_ is the difference in mixed muscle protein‐bound l‐[*ring*‐^2^H_5_]‐phenylalanine enrichment between two timepoints and *E*_*P*_ is the mean precursor enrichment over this time.

Muscle protein net balance (MPNB) was calculated as

(3) $$\text{MPNB}\;\left(\%\cdot h^{-1}\right)=\mathrm{FSR}-\mathrm{FBR}\;$$

where FSR is calculated using the IC pool.

### Statistics

A Student's independent *t*‐test was used to test group differences in subject characteristics, serum insulin concentration AUC, and constants describing arterial enrichment decay. Three‐way ANOVAs with time, group, and immobilization factors were used to detect differences in intracellular free and protein‐bound phenylalanine concentrations and enrichments. Steady state in plasma l‐[*ring*‐^2^H_5_]‐phenylalanine enrichment was determined by linear regression. All other data were analysed using two‐way mixed model ANOVA (time and group factors, or leg and group factors), with Sidak corrections for multiple comparisons applied to follow up post hoc differences. Statistical analysis was performed using GraphPad Prism 8 (GraphPad Software, Inc., San Diego, CA, USA). All data are presented as mean ± SEM with *P* < 0.05 indicating statistical significance. Trends were identified if *P* < 0.10. Outliers were identified if beyond 2 standard deviations from the mean and either re‐analysed (where possible) or omitted.

## Results

### Participant characteristics

There were no significant differences in any of the participants' characteristics between FAST and FED (Table 1). Dietary intakes over the free‐living and immobilized periods were not different between FAST or FED (Table S1). Expressed as total daily intake, only dietary fibre consumption increased by 24 ± 7% during immobilization (*P* < 0.05). As a proportion of daily intake, dietary fat provided 13 ± 6% more energy during the immobilization period in both groups (*P* < 0.05). Total energy, protein, and carbohydrate intake did not differ between free‐living and immobilized periods in either group.

**Table 1:** Participant characteristics of healthy young males undergoing 2 days of unilateral knee immobilization

	FAST	FED
	(*n* = 11)	(*n* = 12)
Age (year)	21 ± 0	22 ± 2
Body mass (kg)	74.5 ± 1.7	72.3 ± 2.6
Height (cm)	180.6 ± 1.8	178.3 ± 1.8
BMI (kg·m^−2^)	22.9 ± 0.8	22.7 ± 0.7
Body fat (% body mass)	11.7 ± 2.2	12.7 ± 3.0

Values represent mean ± SEM. All between treatment comparisons *P* > 0.05.

BMI, body mass index; FAST, saline infusion group; FED, amino acid infusion group.

### Serum insulin concentrations

**Figure 2:**
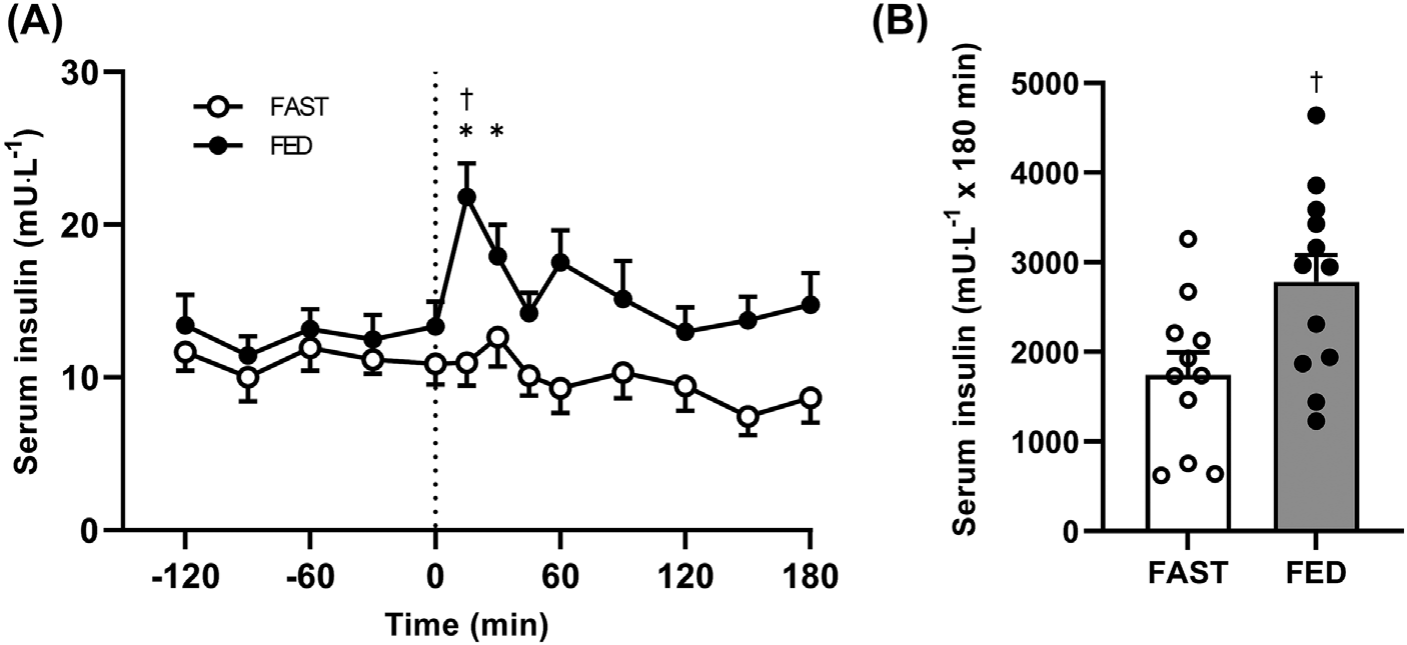
Arterialised serum insulin concentrations. (A) Prior to and during a 3‐h intravenous saline (FAST; *n* = 11) or amino acid (FED; *n* = 12) infusion and (B) total area under the curve (AUC) between 0 and 180 min. Data presented as means ± SEM. Time course data were analysed by two‐way analysis of variance (ANOVA); AUC data compared by unpaired *t*‐test. Significant time by group interaction effect (*P* < 0.001); significant difference between FAST and FED AUC (*P* < 0.05). Group differences in AUC and *post hoc* differences within time by group interaction effects denoted by ^†^ *P* < 0.01 significantly different to FAST at a given time; *P < 0.05 significantly different to 0 min within group.

Prandial infusions significantly increased serum insulin concentrations in FED only (Figure 2; interaction *P* < 0.001) and were overall greater in FED versus FAST (*P* < 0.05). From 0 min, serum insulin increased by 73 ± 14% to peak at 21.8 ± 2.2 mU·L^−1^ at 15 min in FED only (post hoc *P* < 0.001), before returning to baseline at 45 min following the start of the infusion. As a result, the total postprandial area under the curve (AUC) was 60% greater in FED versus FAST (*P* < 0.01).

### Plasma amino acid concentrations

**Figure 3:**
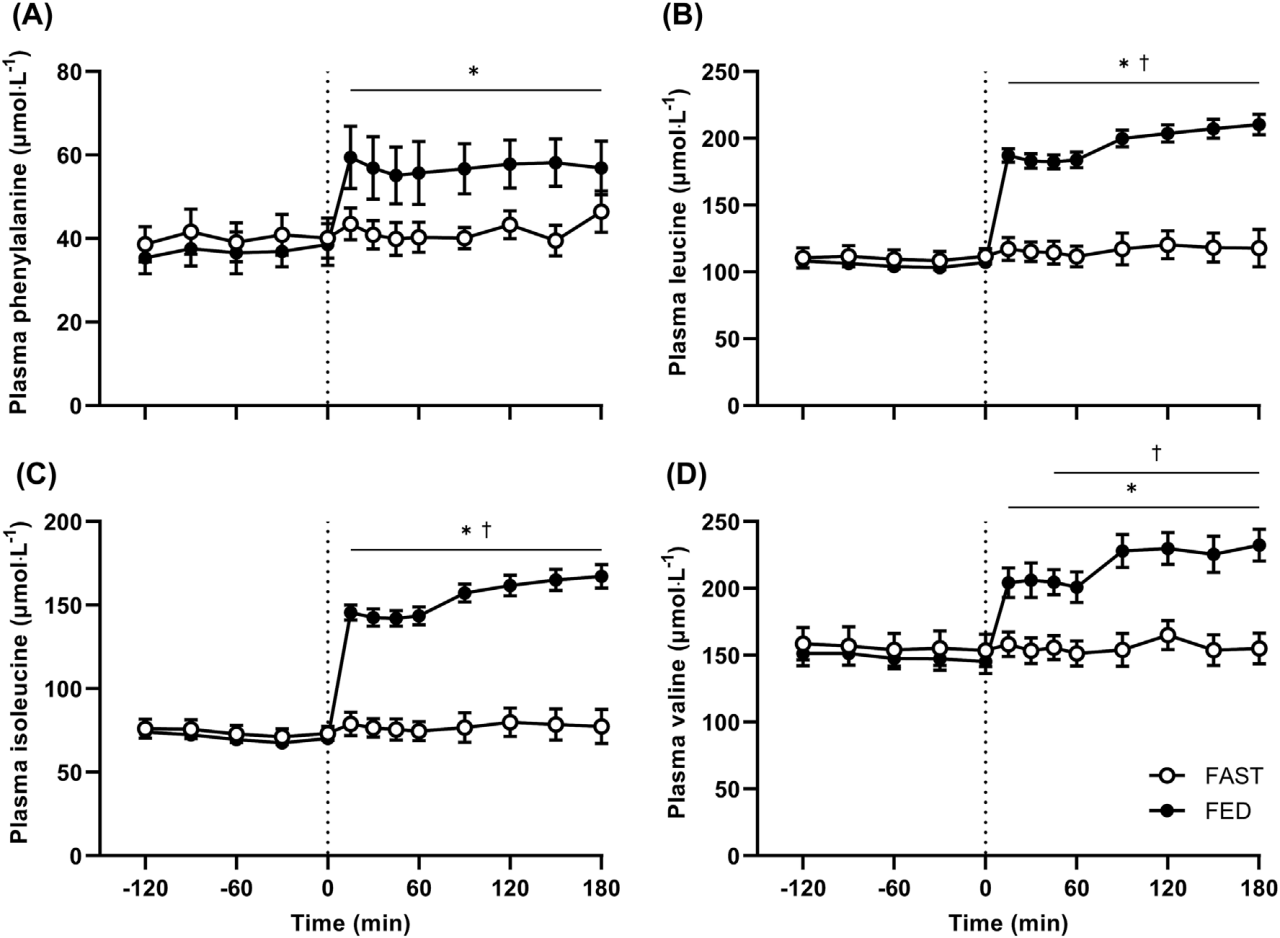
Arterialised plasma. (A) Phenylalanine, (B) leucine, (C) isoleucine, and (D) valine concentrations for 120 min prior to and 180 min during either an intravenous saline (FAST; *n* = 11) or amino acid (FED; *n* = 12) infusion. Data were analysed by two‐way analysis of variance (ANOVA) with time and group factors. Significant time and interaction effects present for all amino acids (*P* < 0.001). Significant group difference in leucine, isoleucine (both *P* < 0.001) and valine (*P* < 0.05). Post hoc differences within interaction effects denoted by ^†^ *P* < 0.05 significantly different to FAST at a given time; *P < 0.05 significantly different to 0 min within group.

Prandial infusions increased plasma phenylalanine concentrations by 59 ± 9% after 15 min in FED only (Figure 3; interaction *P* < 0.001), which remained elevated at all timepoints thereafter (mean increase 53 ± 7%). Concentrations did not differ between any subsequent timepoints thereafter in either group, indicating steady state had been achieved. The mean phenylalanine concentration during prandial infusions in FED was 57 ± 7 μmol·L^−1^ (all post hoc differences at 15 min and beyond *P* > 0.05). Prandial infusions also increased plasma leucine, isoleucine, and valine concentrations at 15 min by 76 ± 5%, 109 ± 7%, and 42 ± 4%, respectively, from 0 min in FED only (all interactions *P* < 0.001). These remained elevated compared with 0 min at all timepoints thereafter (post hoc vs. 0 min *P* < 0.001). Compared with FAST, leucine and isoleucine concentrations were 67 ± 3% and 98 ± 4% greater in FED throughout prandial infusions (post hoc *P* < 0.001), and valine was 41 ± 3% greater from 45 min onwards (post hoc *P* < 0.05). All measured amino acid concentrations remained stable throughout in FAST (all post hoc comparisons *P* > 0.05).

### Muscle amino acid concentrations

Phenylalanine concentrations in the muscle intracellular (IC) pool in IMM were 11 ± 3% and 7 ± 5% greater than CON, in FAST and FED, respectively (immobilization effect *P* < 0.05; Table S2). Prandial infusions increased muscle IC phenylalanine concentration in FED only (time by group interaction *P* < 0.001), an effect independent from immobilization (all immobilization interactions *P* > 0.05). Specifically, muscle IC phenylalanine concentrations in the FED group increased by 23 ± 5% and 37 ± 9% at both 60 and 180 min respectively, versus 0 min (*P* < 0.001). No further increase was observed between 60 and 180 min (*P* = 0.12).

There was no effect of immobilization, group, or time on muscle protein‐bound phenylalanine concentrations (all main effects and interactions *P* > 0.05). Pooled over time, mean concentrations were 0.171 ± 0.007 and 0.175 ± 0 0.011 μmol·mg dry mass (dm)^−1^ in CON and IMM in FAST, and 0.186 ± 0.010 and 0.166 ± 0.008 μmol·mg dm^−1^ in CON and IMM in FED (all main effects and interactions *P* > 0.05).

### Isotopic enrichments

**Figure 4:**
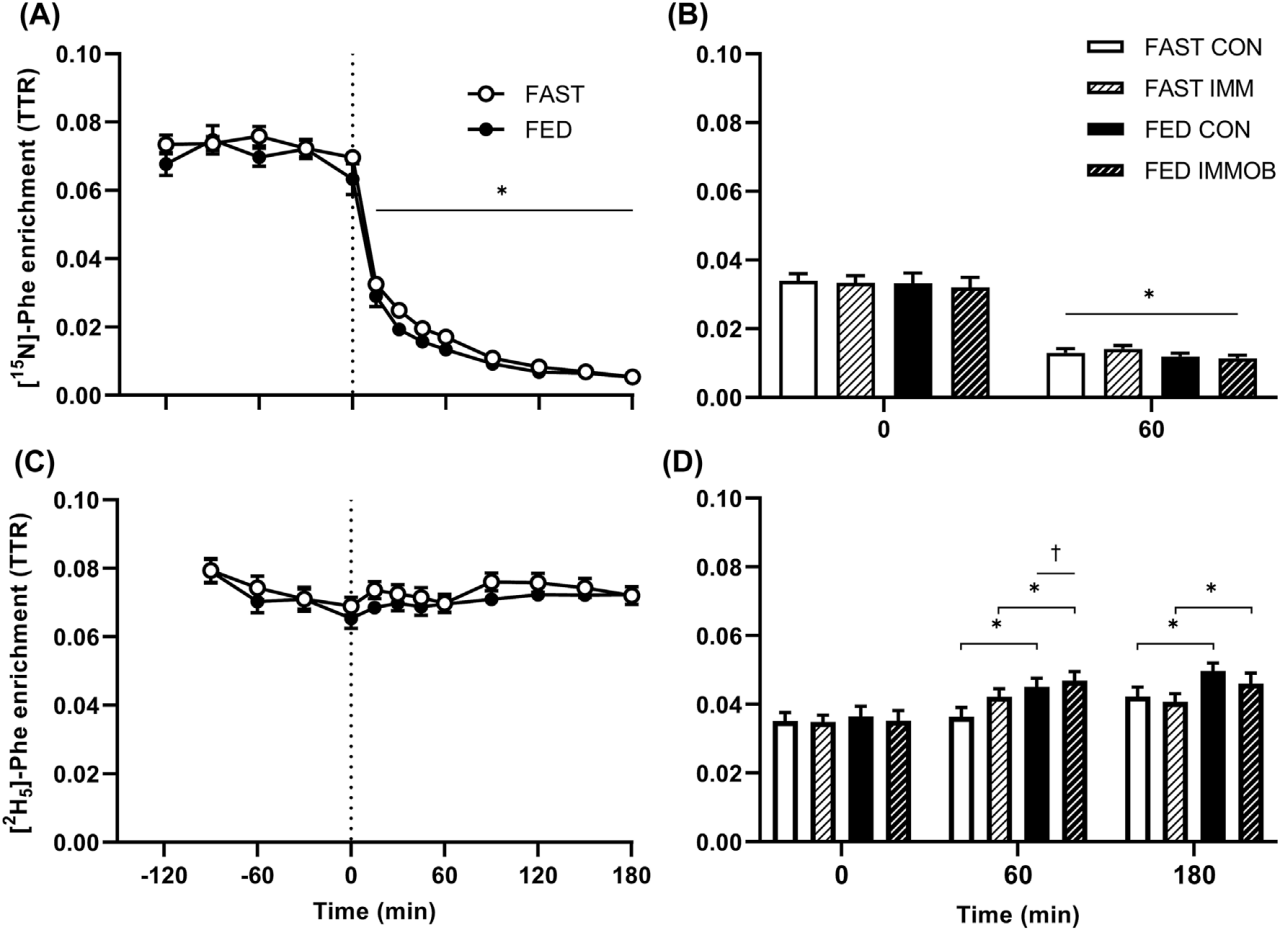
Enrichment of l‐[^15^N]‐phenylalanine in (A) plasma and (B) muscle intracellular (IC) pool, and l‐[*ring*‐^2^H_5_]‐phenylalanine in (C) plasma and (D) IC pool. Time is shown relative to the start of a continuous intravenous saline (FAST; *n* = 11) or amino acid (FED; *n* = 12) infusion, at which time the l‐[^15^N]‐phenylalanine infusion was stopped. A two‐way analysis of variance (ANOVA; time and group factors) and linear regression was used to detect differences in plasma l‐[^15^N]‐phenylalanine and l‐[*ring*‐^2^H_5_]‐phenylalanine enrichment, respectively. A three‐way ANOVA (time, immobilization, and group factors) was used to detect differences in intracellular enrichments, with interactions followed up by two‐way ANOVA. (A) Main effects of group (*P* < 0.05) and time (*P* < 0.001; post hoc differences denoted by *P < 0.001 significantly different to 0 min). (B) Main effects of time (*P* < 0.001; post hoc differences denoted by *P < 0.001 significantly different to 0 min). (C) Plasma l‐[*ring*‐^2^H_5_]‐phenylalanine enrichment remained stable over time (slopes *P* > 0.05 different from 0). (D) Main effects of time (*P* < 0.001). Post hoc difference within immobilization by time interaction (*P* < 0.01) denoted by *P < 0.01 significantly different to 0 min within leg. Post hoc difference within group by time interaction (*P* < 0.05) denoted by ^†^ *P* < 0.05 significantly greater in FED than FAST at given time.

Plasma l‐[^15^N]‐phenylalanine enrichments (Figure 4A) decreased following cessation of l‐[^15^N]‐phenylalanine tracer infusion such that, compared with 0 min, all timepoints thereafter were lower (time effect *P* < 0.001; post hoc at all points *P* < 0.001). Overall, plasma l‐[^15^N]‐phenylalanine enrichments in FED were 11 ± 2% lower than FAST (group *P* < 0.05), but this did not change over time (interaction *P* = 0.52). Intracellular l‐[^15^N]‐phenylalanine enrichments (Figure 4B) decreased by a similar degree in FAST and FED from 0 to 60 min (time *P* < 0.001; group *P* = 0.49). This decrease was not influenced by immobilization (*P* = 0.62), and no interactions were observed (all *P* > 0.05).

Plasma l‐[*ring*‐^2^H_5_]‐phenylalanine enrichments (Figure 4C) remained stable throughout infusion as determined by linear regression in both FAST and FED (*P* = 0.64 and *P* = 0.67, respectively, for slope deviating from zero). l‐[*ring*‐^2^H_5_]‐phenylalanine enrichments in the muscle IC pool (Figure 4D) increased from basal (time effect; *P* < 0.001) at both 60 and 180 min (group*time interaction *P* < 0.05; immobilization*time interaction *P* < 0.01), and were greater in FED compared with FAST at 60 min (legs pooled, group*time post hoc *P* < 0.05). With muscle IC enrichments pooled across groups, further increases between 60 and 180 min in CON (immobilization by time post hoc *P* < 0.01), but not in IMM (immobilization*time post hoc *P* = 0.87), were observed.

Protein‐bound l‐[*ring*‐^2^H_5_]‐phenylalanine enrichments (Table S3) increased over time (*P* < 0.001) and were greater in FED (*P* < 0.05). The increase over time was influenced by immobilization (interaction *P* < 0.01), but while significant differences were observed between all timepoints in CON (post hoc *P* < 0.001) and in IMM (post hoc *P* < 0.05), no post hoc differences between legs were observed at any timepoint.

### Fractional breakdown rates

**Figure 5:**
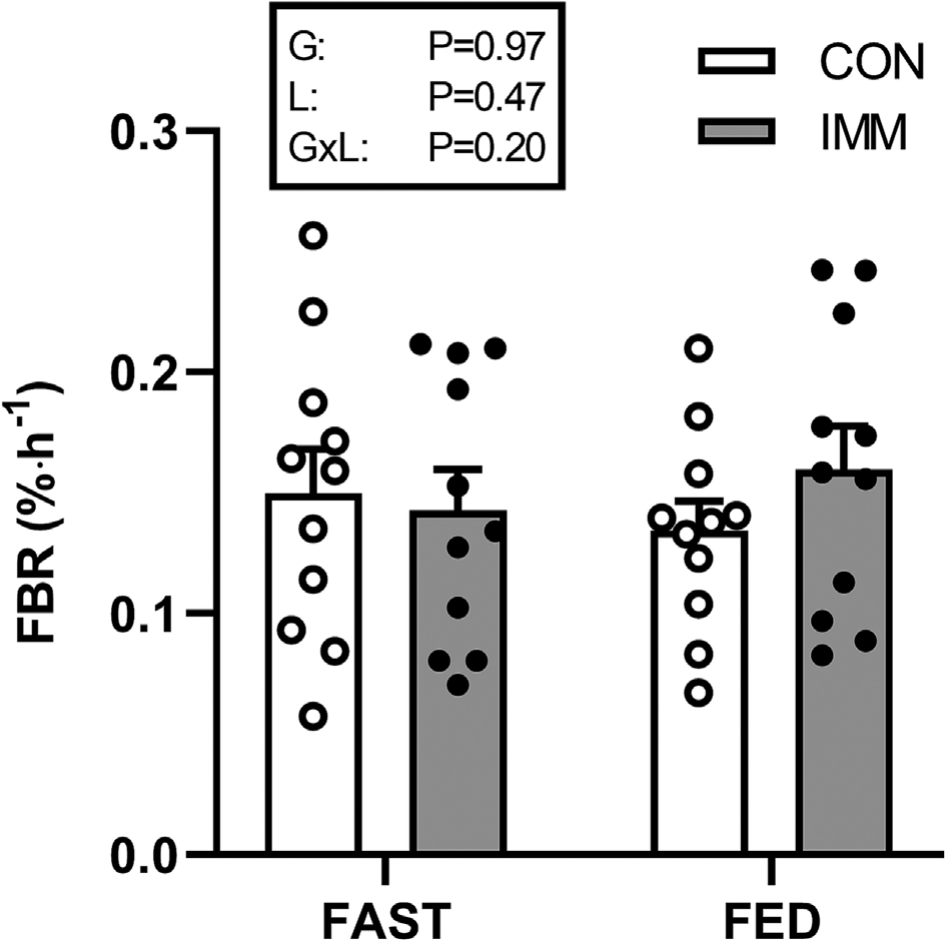
Fractional breakdown rate (FBR) of mixed muscle proteins in control (CON; open symbols) and immobilized (IMM; filled symbols) legs during intravenous saline (FAST; *n* = 11) or amino acid (FED; *n* = 11) infusions. Data were analysed by two‐way analysis of variance (ANOVA). All differences *P* > 0.05.

Muscle protein FBRs (Figure 5) are depicted in *n* = 11 for both FAST and FED, as one participant was considered an outlier (values >3 SD from the mean), and having insufficient tissue yield for re‐analysis. Both *Q*_*M*_ */T* and *P* were determined from the muscle sample at 60 min, with the latter from the l‐[*ring*‐^2^H_5_]‐phenylalanine enrichment, as intracellular phenylalanine concentration and the ratio of inward transport to breakdown increased with prandial infusions in FED (Figure 4D; Table S4). All parameters used to calculate FBR are presented in Table S4. Immobilization did not affect FBR in FAST (CON: 0.150 ± 0.018%·h^−1^; IMM: 0.143 ± 0.017%·h^−1^) or FED (CON: 0.134 ± 0.012%·h^−1^; IMM: 0.160 ± 0.018%·h^−1^; immobilization effect *P* = 0.47). Moreover, prandial infusions did not affect FBR (group effect *P* = 0.97) and nor was this modulated by immobilization (interaction *P* = 0.20).

### Fractional synthesis rates

**Figure 6:**
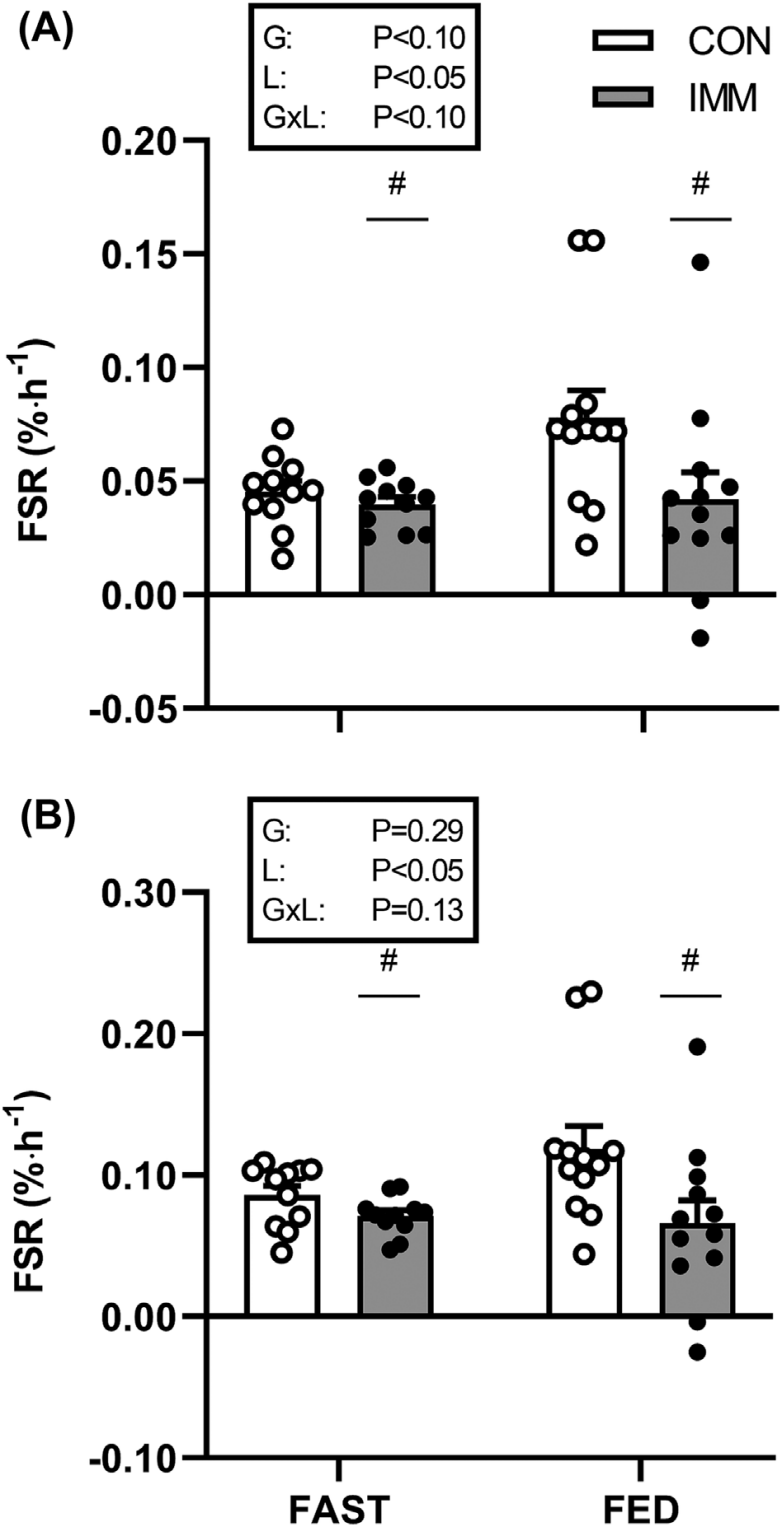
Mixed muscle protein fractional synthesis rates (FSR) calculated from (A) plasma and (B) intracellular precursor pools in control (CON; open symbols) and immobilized legs (IMM; filled symbols) during intravenous saline (FAST; *n* = 11) or amino acid infusion (FED; *n* = 12). Data were analysed by two‐way analysis of variance (ANOVA). Main effect of immobilization denoted by ^#^ *P* < 0.05 significantly different to CON. (A) Trend for immobilization by group interaction observed (*P* < 0.1).

Muscle protein FSRs during the prandial infusions trended toward being greater in FED than FAST, when calculated from the plasma precursor only (Figure 6A; *P* < 0.10). Immobilization decreased muscle protein FSR calculated from both the plasma and IC precursors (Figure 6A and B, respectively; both *P* < 0.05). Based on the plasma precursor, there was a trend for the immobilization induced decrease to be greater in the FED versus FAST groups (−35 ± 18% vs. ‐4 ± 10%, respectively; interaction *P* < 0.10).

### Muscle protein net balance

**Figure 7:**
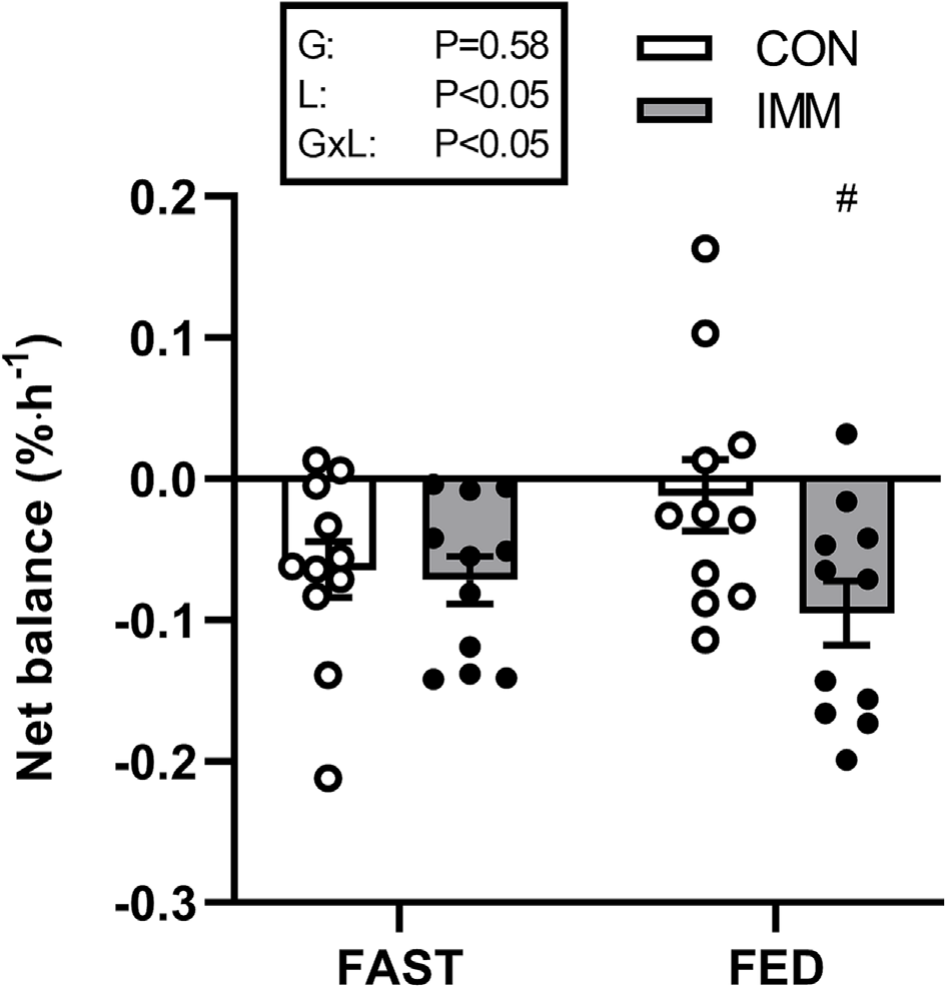
Mixed muscle protein net balance in control (CON; open symbols) and immobilized legs (IMM; filled symbols) during intravenous saline (FAST; *n* = 11) or amino acid infusion (FED; *n* = 11). Data were analysed by two‐way analysis of variance (ANOVA). Main effect of immobilization (*P* < 0.05) and immobilization by time interaction (*P* < 0.05). Post hoc differences within interaction denoted by ^#^ *P* < 0.01 significantly different to contralateral CON leg.

There was no overall effect of prandial infusions on muscle protein net balance (MPNB; group *P* > 0.05; Figure 7). However, immobilization impaired MPNB in both FAST and FED (*P* < 0.05). Moreover, the effect of immobilization was exacerbated in FED (interaction *P* < 0.05). These differences were present regardless of precursor choice.

## Discussion

In the present study we show the novel finding that, contrary to our hypothesis, short‐term (2 days) lower limb immobilization does not modulate *in vivo* postabsorptive or simulated postprandial rates of muscle protein breakdown (MPB), measured dynamically and directly as fractional breakdown rates (FBR) in humans. In parallel, we also show for the first time that merely 2 days of immobilization is sufficient to modestly lower postabsorptive muscle protein synthesis (MPS) and induce anabolic resistance to elevations in circulating amino acids. Consequently, we report a comprehensive dataset to show muscle protein net balance is reduced after only 2 days of disuse, an effect driven by blunted MPS rates and most pronounced under postprandial compared with postabsorptive conditions.

We^5^, ^14^ and others^8^, ^9^, ^10^, ^11^, ^12^ have suggested that the decrease in postabsorptive and postprandial MPS primarily drives muscle disuse atrophy. However, cumulative rates of MPS measured previously over the first 2 days of immobilization cannot fully explain the observed changes in muscle volume.^13^ While these calculations were conducted assuming that changes in muscle volume are reflective of changes in muscle protein pool size (amongst other assumptions discussed elsewhere^13^) the validity of these assumptions is unconfirmed to date, which leaves open the possibility that increased MPB also contributes to early‐stage disuse atrophy. Here, we show for the first time that short‐term immobilization does not increase MPB when measured directly and dynamically as FBR (Figure 5). In our approach we accounted for tracer dilution rates, differences in the intracellular (IC) pool size (i.e., a component of *Qm/T* in equation *1*; increased by ~6% after 2 days (Supplemental Table 2) and ~30% after 14 days^12^ of immobilization), as well as the relative contribution between MPB and arterial inward transport to IC tracer decay (i.e., the constant *P* in equation *1*), which altogether allowed us to quantify FBR. These findings are supported by a recent study that determined tracer dilution rates in the muscle IC pool and showed that postabsorptive IC tracer dilution rates, to which MPB is a contributing factor, were not affected by 4 days of unilateral knee immobilization.^17^ Moreover, to our knowledge no study to date has investigated the effect of immobilization on MPB in the postprandial state, despite the well‐established role of exogenous amino acids in creating a net positive protein balance^30^, ^31^ and demonstrated anabolic resistance of MPS with immobilization.^5^, ^12^ This is largely due to methodological concerns, as bolus feeding interrupts the physiological steady state that is assumed when directly measuring FBR.^23^, ^32^ To satisfy this assumption we used an innovative study design to quantify postprandial FBR in the present study, which was achieved by primed continuous intravenous amino acid infusion. As such, we demonstrate comprehensively that short‐term immobilization does not increase MPB in either the postabsorptive or a simulated postprandial state, providing compelling evidence that short‐term disuse‐induced atrophy is not driven in any major part by elevated MPB.

In contrast to the lack of effect of knee immobilization on MPB, here we demonstrate that MPS was suppressed by ~20–25% following short‐term immobilization (Figure 6). These findings extend on previous work by showing that the immobilization‐induced suppression of MPS already occurs much earlier (i.e., following merely 2 days of disuse) than previously shown (4–5 days^5^, ^17^). Moreover, there was a trend for greater impairment of MPS in the postprandial than the postabsorptive state (~35% vs. ~4% respectively; Figure 6A). While this effect was only observed when calculated from the more stable plasma precursor pool, these data suggest the ‘anabolic resistance’ that has been categorized after 5–14 days of disuse seemingly manifests immediately after the onset of immobilization. Our recent work suggests that anabolic resistance must reside between an amino acid leaving the circulation and its committal to protein synthesis.^33^ The within‐participants design used here precludes any systemic factors from causing this, under the assumption that both the control and immobilized legs were exposed to the same systemic milieu (i.e., elevated circulating insulin and amino acid concentrations, Figures 2 and 3). Moreover, the prandial amino acid infusion increased intracellular phenylalanine concentrations (~20% increase after 60 min, Table S2) and l‐[*ring*‐^2^H_5_]‐phenylalanine enrichments (Figure 4D) equally between control and immobilized legs in the absence of differences in FBR (Figure 5), providing further evidence that immobilization does not affect muscle amino acid uptake into the muscle cell in this simulated‐fed state. Despite unchanged (Figure 3) or even increased^12^ intracellular amino acid concentrations observed following disuse, static molecular markers have indicated impaired protein phosphorylation status of proteins in the Akt‐mTOR‐p70S6k pathway in response to elevated amino acid concentrations.^5^, ^12^, ^34^ Although the mechanistic underpinning of anabolic resistance requires further investigation, it could imply that anabolic resistance is located on this signalling pathway, making this a potential target for designing effective interventions. Given the rapid development of insulin resistance reported in the same timeframe,^35^ the absence of muscle contraction is likely a key contributor to disuse‐induced anabolic resistance. As such, re‐introducing muscle contraction (and therefore signalling through the Akt‐mTOR‐p70S6k pathway^36^) could be one of the most potent stimuli to maintain muscle mass and metabolic health during disuse.

As a consequence of impaired MPS with no concurrent changes in MPB, here we show 2 days of unilateral knee immobilization resulted in more negative net muscle protein balance in both the postabsorptive and simulated postprandial states, when compared with a control leg (Figure 7). If we assume, as others have done,^1^ that 9 h per day are spent in the postprandial state, this would account for ~0.86%·day^−1^ more atrophy in the immobilized than control leg, which is remarkably consistent with our previously measured rates of atrophy of ~0.85%·day^−1^ using the same immobilization model.^18^ Muscle protein net balance was decidedly more negative with immobilization compared with the control leg in the simulated postprandial state, accounting for ~7‐fold more muscle protein loss than during postabsorptive periods. This would support previous suggestions that impaired postprandial MPS (i.e., anabolic resistance), rather than changes in MPB, is a major factor underpinning muscle disuse atrophy.^1^, ^37^ These findings also extend on our previous work in which we indirectly calculated the contribution of MPS to changes in muscle volume, and estimated that immobilization‐induced declines in cumulative (myofibrillar) MPS explain ~11 g of a total 42 g loss in muscle mass.^13^ We underline the need for caution when using indirect calculations (involving various assumptions, including that changes in muscle volume or cross‐sectional area reflect changes in muscle protein pool size^13^) to determine how muscle protein synthesis and breakdown rates may reflect changes in muscle mass, and *vice versa*, particularly when measuring only one component of this calculation directly. Importantly, until the unknowns in those calculations have been eliminated, there is a clear need to rely on the more robust, directly measured outcomes (e.g., MPS/MPB and/or measures of muscle mass) and show prudence when making consequent, indirect assumptions.

Rather than provide a protein or amino acid bolus, which would violate assumptions of a physiological steady state when calculating FBR, we simulated postprandial conditions using a primed continuous amino acid infusion. Approximately 15 g of amino acids were infused in the 3‐h simulated postprandial condition, supplying a comparable quantity of exogenous amino acids to what may be expected after consuming ~30 g of whey or milk protein, after accounting for losses from incomplete absorption and amino acids retained in splanchnic tissues.^38^ Importantly, systemic insulin and amino acid concentrations were comparable to those observed after such a dose, which has been demonstrated to maximally stimulate MPS.^39^ Indeed, amino acid infusions ranging from 44 to 162 mg·kg^−1^ body mass^−1^·h^−1^ have been used to recreate a postprandial anabolic stimulus, robustly stimulating MPS by ~30–70%.^12^, ^40^, ^41^ In accordance, we observed a trend for 3 h FSR to be greater with amino acid infusion (67.5 mg·kg^−1^·h^−1^ used presently), when calculated from the plasma precursor, versus in the postabsorptive state. Although this was limited to a trend, we suspect that the absence of a clear effect on MPS is due to our between‐groups design (which was necessary to address our primary research question focused on FBR), as numerically, in the control leg FSR was ~72% greater with amino acid than with saline infusion. Indeed, prior work showing the stimulatory effects of amino acid infusion has quantified MPS immediately following a postabsorptive period within the same participants.^12^, ^40^, ^41^

Rates of MPB are often considered to be as sensitive (albeit less responsive in magnitude) as MPS in response to nutritional stimuli, readily falling in tandem with rising MPS to contribute to a postprandial net muscle protein gain.^1^ While MPS responded in line with previous work, unexpectedly, we report that amino acid infusion (and associated hyperinsulinaemia) did not influence rates of MPB (~0.15%·h^−1^ in both postabsorptive and simulated postprandial conditions, pooled across legs; Figure 5). Indeed, 2‐pool modelling of amino acid kinetics across a limb routinely demonstrates that modest physiological insulin and/or amino acid infusions, as well as ingestion of amino acid and protein‐based beverages, suppress rate of amino acid appearance from muscle protein breakdown by ~15–50%.^42^, ^43^, ^44^ However, 3‐pool models, which take into account amino acid kinetics within the muscle cell, show that intracellular appearance from breakdown is less readily suppressed by (simulated) feeding interventions, if at all.^30^, ^31^ Moreover, these models rely on the assumption that muscle IC pool size remains constant with feeding, which we (Table S2) and others^41^, ^45^ demonstrate not to be the case. Regardless, the present study builds on data from 3‐pool models to reveal an intriguing possibility that a typical anabolic stimulus does not potently suppress rates of MPB *per se* but exerts its anabolic effect primarily via stimulation of MPS. With this in mind, future work is required to comprehensively investigate whether MPB is readily manipulated by oral protein intake both in free‐living conditions and following disuse.

We conclude that short‐term muscle disuse does not affect MPB when measured directly and dynamically in either the postabsorptive or simulated postprandial states. Given that immobilization reduced basal MPS rates and induced anabolic resistance to amino acid provision, our data clearly show that muscle protein loss (i.e., sustained negative muscle protein balance) associated with brief periods of disuse in otherwise healthy individuals is driven near exclusively by a suppression in MPS. Given the decline in muscle protein net balance was most profound in the postprandial state, we propose that interventions aimed at mitigating short‐term muscle disuse atrophy should primarily focus on sensitizing the muscle tissue to the anabolic properties of amino acids (e.g., muscle contraction).

## Funding

This work was funded by a grant from Nutricia Research Foundation (2019‐30) held by M.L.D. G.F.P. was funded by a PhD studentship from the University of Exeter and Beachbody LLC held by F.B.S. A.J.M. and D.R.A. are supported in part by National Institute of Aging Grant P30‐AG024832.

## Conflict of interest

No conflicts of interest are declared.

## Supporting information


**Table S1.** Dietary intake as assessed by weighed food diary during a 3‐day free living period and during 2 days of unilateral knee immobilization.
**Table S2.** Intracellular phenylalanine concentration in dry muscle (nmol·mg ^−1^).
**Table S3.** Mixed muscle protein‐bound L‐[*ring*‐^2^H_5_]‐phenylalanine enrichment (TTR%) during primed continuous L‐[*ring*‐^2^H_5_]‐phenylalanine infusion.
**Table S4.** Parameters used in calculation of FBR from a single biopsy timepoint.
