# Supplementary material for: Short‐term disuse does not affect postabsorptive or postprandial muscle protein fractional breakdown rates
Source: J Cachexia Sarcopenia Muscle. 2023 Jul 11;14(5):2064–75. doi: 10.1002/jcsm.13284 (PMC10570083; doi:10.1002/jcsm.13284)
Supplement: Supplementary file 1 — Table S1. Dietary intake as assessed by weighed food diary during a 3‐day free living period and during 2 days of unilateral knee immobilization. Table S2. Intracellular phenylalanine concentration in dry muscle (nmol·mg −1). Table S3. Mixed muscle protein‐bound L‐[ring‐2H5]‐phenylalanine enrichment (TTR%) during primed continuous L‐[ring‐2H5]‐phenylalanine infusion. Table S4. Parameters used in calculation of FBR from a single biopsy timepoint. [file JCSM-14-2064-s001.docx]

**Supplemental information**

**Supplemental Methods**

Immobilization

Unilateral knee immobilization was achieved using a leg brace (X-ACT Donjoy brace; DJO Global, Carlsbad, CA, USA) according to methods previously used by our laboratory [S1] allowing for the non-immobilized leg to act as a within-subject control. Participants ambulated using crutches after receiving instructions, with the knee fixed at an angle of 70° flexion (full knee extension considered as 0°) to remove the ability to bear weight. Subjects were asked to refrain from ground contact and muscle contraction, apart from twice-daily ankle rotation exercises to aid venous return. Adhesive tape with the experimenter’s signature inscribed was wrapped around adjustable parts of the brace such that any alterations to the brace would be identifiable and result in exclusion from the study; no adjustments were identified and thus no participants were excluded. Participants were provided a plastic cover to wear when showering to reduce disruption to daily living activities.

Nutritional intake

All participants were instructed how to complete a weighed diet diary and were asked to record 2 weekdays and 1 weekend day of their habitual, free-living diet prior to the immobilization period. During the 48 h immobilization period, a second diet diary was completed. On the evening prior to the test day, participants were provided a standardized meal (976 kcal, 38.4% energy (En%) carbohydrate, 16.8 En% protein, 44.8 En% fat) and were instructed to remain fasted thereafter until the completion of their visit to the laboratory the next day.

**Supplemental Table 1**: Dietary intake as assessed by weighed food diary during a 3-day free living period and during 2 days of unilateral knee immobilisation.

|  | **FAST** | | **FED** | |
| --- | --- | --- | --- | --- |
|  | **Free-living** | **Immobilisation** | **Free-living** | **Immobilisation** |
| **Energy (MJ·d^-1^)** | 9.0 ± 1.3 | 10.5 ± 1.4 | 7.8 ± 0.8 | 8.2 ± 0.7 |
| **Protein (g·kg bm^-1^·d^-1^)** | 1.28 ± 0.16 | 1.40 ± 0.14 | 1.18 ± 0.16 | 1.17 ± 0.11 |
| **Protein (g·d^-1^)** | 95 ± 11 | 105 ± 12 | 86 ± 12 | 84 ± 8 |
| **Carbohydrates (g·d^-1^)** | 230 ± 37 | 280 ± 47 | 212 ± 17 | 225 ± 16 |
| **Fat (g·d^-1^)** | 87 ± 17 | 108 ± 14 | 74 ± 11 | 81 ± 8 |
| **Fibre (g·d^-1^)** | 24 ± 4 | 24 ± 3* | 17 ± 2 | 21 ± 2* |
| **Protein (En%)** | 19 ± 1 | 18 ± 2 | 19 ± 2 | 17 ± 1 |
| **Carbohydrates (En%)** | 43 ± 3 | 44 ± 3 | 48 ± 3 | 47 ± 2 |
| **Fat (En%)** | 35 ± 3 | 38 ± 2* | 33 ± 2 | 36 ± 1* |
| **Fibre (En%)** | 2 ± 0 | 2 ± 0 | 2 ± 0 | 2 ± 0 |

Values represent mean ± SEM. FAST, saline infusion group; FED, amino acid infusion group. Data analysed with 2-way ANOVA. Significant main effect of immobilised versus free-living periods presented as: **P*<0.05 different to free-living.

**Supplemental Table 2**: Intracellular phenylalanine concentration in dry muscle (nmol∙mg ^-1^).

|  | **FAST** | | **FED** | |
| --- | --- | --- | --- | --- |
|  | **CON** | **IMM** | **CON** | **IMM** |
| **0 min** | 0.187 ± 0.017 | 0.210 ± 0.014 | 0.180 ± 0.012 | 0.193 ± 0.015 |
| **60 min** | 0.197 ± 0.014 | 0.207 ± 0.016 | 0.225 ± 0.014* | 0.222 ± 0.011* |
| **180 min** | 0.193 ± 0.015 | 0.207 ± 0.014 | 0.233 ± 0.009*^†^ | 0.253 ± 0.014*^†^ |

Values are mean ± SEM. FAST, saline infusion group; FED, amino acid infusion group; CON, control leg; IMM, immobilised leg. Data were analysed with 3-way ANOVA. Significant differences between CON and IMM (*P*<0.05). Time and time by group interaction (*P*<0.001). Post hoc difference within time by group interaction denoted by *P<0.001 significantly greater than 0 min in FED only; ^†^*P*<0.01 significantly greater in FED than FAST at given timepoint.

**Supplemental Table 3**: Mixed muscle protein-bound L-[*ring*-^2^H_5_]-phenylalanine enrichment (TTR%) during primed continuous L-[*ring*-^2^H_5_]-phenylalanine infusion.

|  | **FAST** | | **FED** | |
| --- | --- | --- | --- | --- |
|  | **CON** | **IMM** | **CON** | **IMM** |
| **0 min** | 0.003 ± 0.001 | 0.002 ± 0.001 | 0.006 ± 0.001 | 0.006 ± 0.002 |
| **60 min** | 0.007 ± 0.001* | 0.007 ± 0.001* | 0.011 ± 0.002* | 0.011 ± 0.003* |
| **180 min** | 0.013 ± 0.002* | 0.011 ± 0.001* | 0.022 ± 0.003* | 0.016 ± 0.003* |

Values are mean ± SEM. FAST, saline infusion group; FED, amino acid infusion group; CON, control leg; IMM, immobilised leg. Data were analysed with 3-way ANOVA. Significant differences between FAST and FED (*P*<0.05). Time and time by immobilisation interaction present (*P*<0.05). Post hoc difference within immobilisation by time interaction denoted by *P<0.001 significantly different to 0 min within leg (groups pooled).

**Supplemental Table 4**: Parameters used in calculation of FBR from a single biopsy timepoint.

|  | **FAST** | | **FED** | | |
| --- | --- | --- | --- | --- | --- |
| *l* | -0.017 ± 0.001 | | -0.017 ± 0.001 | | |
| *a* | 0.027 ± 0.002 | | 0.035 ± 0.002* | | |
| *b* | 0.043 ± 0.001 | | 0.033 ± 0.001* | | |
| *k* | -0.529 ± 0.006 | | -0.525 ± 0.004 | | |
|  |  |  | |  |  |
|  | *CON* | *IMM* | | *CON* | *IMM* |
| *Em(t) (TTR)* | 0.013 ± 0.001 | 0.014 ± 0.001 | | 0.012 ± 0.001 | 0.011 ± 0.001 |
| *Qm/T* | 0.0011 ± 0.0001 | 0.0012 ± 0.0001 | | 0.0012 ± 0.0000 | 0.0013 ± 0.0001 |
| *P* | 1.4 ± 0.4 | 1.9 ± 0.3 | | 2.0 ± 0.2 | 2.2 ± 0.3 |
| *t (min)* | 62 ± 1 | 62 ± 1 | | 62 ± 1 | 62 ± 1 |

Data presented as mean ± SEM. *CON*; control leg. *IMM*; immobilised leg. The constants *l, a, b, k* are derived from the decay of arterial L-[^15^N]-phenylalanine enrichment (see equations derived by [S2]). *Em(t)* is the L-[^15^N]-phenylalanine enrichment expressed as tracer-to-tracee ratio (TTR) in the intracellular pool at time *t* (min). *Qm/T* is the ratio of total phenylalanine in the intracellular (*Qm*) and bound pools (*T*), and *P* is ratio of tracee from artery to that coming from breakdown, calculated from plasma and intracellular L-[*ring*-^2^H_5_]-phenylalanine enrichment at time *t*. Constants *l, a, b, k* analysed by unpaired t-test, with significance denoted by **P*<0.05 different to FAST. *Em(t), T/Qm, P* and *t* analysed by 2-way ANOVA; trend for feeding by immobilisation interaction present for *Em(t)* (*P*=0.06), but all other comparisons not significant (*P*>0.05).

**Supplementary references**

S1. Kilroe SP, Fulford J, Jackman SR, van Loon LJ, Wall BT. Temporal muscle-specific disuse atrophy during one week of leg immobilization. Med Sci Sports Exerc. 2020;52:944-54. doi:10.1249/MSS.0000000000002200

S2. Zhang XJ, Chinkes DL, Sakurai Y, Wolfe RR. An isotopic method for measurement of muscle protein fractional breakdown rate in vivo. Am J Physiol. 1996;270:E759-67. doi:10.1152/ajpendo.1996.270.5.E759
